# Identification of genes that contribute to drought stress tolerance in *Populus*

**DOI:** 10.1186/1753-6561-5-S7-P79

**Published:** 2011-09-13

**Authors:** Muhammad Arshad, Kamal Biswas, Jim Mattsson, Sherryl Bisgrove, Aine Plant

**Affiliations:** 1 Department of Biological Sciences, Simon Fraser University, Burnaby, BC, Canada, V5A 1S6

## Background

The cultivation of poplars (*Populus* spp.) is favored for forestry and reclamation purposes all over the northern hemisphere where they represent a commercially important resource. Poplars may become a component of programs to optimize carbon sequestration however; poplars are generally regarded as drought sensitive. The patterns of episodic drought over the last decade suggest that the development of drought tolerant poplar genotypes could be a useful tool to achieve sustained forest productivity [1]. Previous reports have shown that expression of hundreds of poplar genes changes in response to drought, presenting a problem in the identification of genes that are more important than others in counteracting the harmful effects of drought [2,3].

The genus *Populus* contains many fast growing hybrids that show varied drought tolerance according to genotype [4]. Hence, there is genetic variation among poplar hybrids that can be used to identify genes that contribute to drought stress tolerance. Despite extensive physiological and morphological descriptions of the response of Populus to drought, little work has been undertaken to explain genotype differences at the gene level. Therefore, this research has been undertaken and its major objective is to identify the genes that contribute to drought stress tolerance in poplar by correlating the physiological responses to gene expression. These genes may potentially be used as molecular markers in the drought tolerance breeding programs.

## Materials and methods

Hardwood cuttings of 9 poplar hybrids (Green Giant, Assiniboine, AP-36, Canam, Katepwa, Hill, Walker, WP-69 and WP-86) were obtained from Dr. Barb Thomas (Alberta Pacific Forest Industries) and Bill Schroeder (Agriculture & Agri Food Canada, Indian Head, Saskatchewan). Cuttings were established in the greenhouse and plants were obtained for the drought stress trial. At the start of the trial, water was withheld from plants used for drought experiment whereas control plants were watered regularly. During the drought stress trial, a plastochron index was established that helped to collect the data from similar leaves of all drought stressed and control plants. Data were collected from 9 different poplar hybrids at 3 time points (mild stress, severe stress and recovery). Split plot design was used to collect the data from drought stressed and control plants using genotypes as main plots (9) and time points as sub-plots (3). Physiological data were collected for height growth, new leaf formation, water potential (ψ) and relative water content (RWC) from drought stressed and control plants. Young leaves were also collected for gene expression analysis from both stressed and control plants. Quantitative polymerase chain reaction (Q-PCR) was used to analyze the expression of several candidate drought responsive genes.

## Results

Drought significantly affected the growth of the trees (Figure 1). Data from 3 drought stress trials in the greenhouse showed that different hybrids responded drought stress differently. Some hybrids behaved as tolerant whereas others as sensitive and some hybrids showed intermediate response to drought. By statistical analysis of physiological data, we ranked these 9 poplar hybrids according to their drought tolerance ability. Statistical analysis showed that Walker maintained significantly better height growth, leaf formation, ψ and RWC during mild, severe stress and recovery as compared to all other hybrids; therefore Walker was identified as the most drought tolerant hybrid genotype. On contrary, Green Giant showed poor maintenance of physiological parameters at all time points and hence was regarded as the least drought tolerant hybrid genotype. An intermediate response of all other hybrids was recorded during 3 drought stress trials in the greenhouse. Further, we focused only on 2 genotypes (the most and the least drought tolerant) to analyze the expression of several candidate drought responsive genes, which were obtained from microarray studies in poplar. Q-PCR results revealed that selected 2 genotypes had no significant effect on the stress response of some genes, however, other genes showed marked and significant differences in expression between the two genotypes.

## Conclusions

• Three trials in the greenhouse showed that physiological response to drought varies among different poplar hybrids.

• Walker was identified as the most whereas Green Giant as the least drought tolerant genotype among a group of poplar hybrids.

• Q-PCR analysis showed that among a set of candidate drought responsive genes, some genes showed differential expression between the most (Walker) and the least (Green Giant) drought tolerant genotypes.

• The genes showing differential expression in the most and the least drought tolerant genotypes might be playing an important role in drought tolerance of poplar..

• We have identified *Populus* genes those expression correlate with drought tolerance, providing candidate genes for drought tolerance breeding.

• These genes may also be used as molecular markers for drought tolerance in *Populus*.

## References

[B1] Rood S Braatne J Hughes F Ecophysiology of riparian cottonwoods: stream flow dependency, water relations and restoration Tree physiol 2003 23 1113 1124 14522717 10.1093/treephys/23.16.1113

[B2] Plomion C Lalanne C Claverol S Meddour H Kohler A Bogeat-Triboulot M Barre A Provost G.L Dumazet H Jacob D Bastien C Dreyer E Daruvar A Guehl J Schmitter J Martin F Bonneu M Mapping the proteome of poplar and application to the discovery of drought-stress responsive proteins Proteomics 2006 6 6509 6527 10.1002/pmic.200600362 17163438

[B3] Street N Skogstrom O Sjodin A Tucker J Acosta MR Nilsson P Jansson S Taylor G The genetics and genomics of the drought response in *Populus* The Plant J 2006 48 321 341 10.1111/j.1365-313X.2006.02864.x 17005011

[B4] Marron N Villar M Dreyer E Delay D Boudouresque E Petit J Delmotte F Guehl J Brignolas F Diversity of leaf traits related to productivity in 31 Populus deltoides × Populus nigra clones Tree Physiol 2005 25 425 435 15687091 10.1093/treephys/25.4.425

